# Supplementary material for: Seismic events miss important kinematically governed grain scale mechanisms during shear failure of porous rock
Source: Nat Commun. 2022 Oct 18;13:6169. doi: 10.1038/s41467-022-33855-z (PMC9579157; doi:10.1038/s41467-022-33855-z)
Supplement: Supplementary file 2 — Supplementary Information [file 41467_2022_33855_MOESM2_ESM.docx]

Supplementary Information

Seismic events miss important kinematically governed grain scale mechanisms during shear failure of porous rock

A. Cartwright-Taylor, M.-D. Mangriotis, I. G. Main, I. B. Butler, F. Fusseis, M. Ling, E. Andò, A. Curtis, A. F. Bell, A. Crippen, R. E. Rizzo, S. Marti, D. D. Leung, O. V. Magdysyuk

Correspondence to: [alexis.cartwright-taylor@ed.ac.uk](mailto:alexis.cartwright-taylor@ed.ac.uk)

**This PDF file includes:**

Supplementary Note 1: Feedback control of deformation

Supplementary Figs. 1 to 17

Supplementary Tables 1 to 2

Supplementary References

**Separate supplementary files for this manuscript:**

Supplementary Movies 1 to 9

Supplementary Note 1

Feedback control of deformation

Our feedback control system allowed us to control the actuator of our rock press, according to the AE event rate, actuator displacement and the applied axial stress. It is based on the software-controllable, ultra-high pressure Cetoni Nemesys XL syringe pump that drives the hydraulic actuator. Two analogue inputs to the pump controller were used to capture the actuator pressure and displacement. The ASC monitoring system provides a digital signal output, which outputs a pulse when an AE event is detected above a pre-defined amplitude threshold. The output is interfaced to our control system via an Arduino microcontroller board linked to the control PC over a serial interface. Each pulse report is a pair *(N, dt)* where *dt* is the time since the last report and *N* is the number of pulses seen during that interval. Any report where *N* > 0 is treated as a single AE event (i.e., *N* = 1), and for each pulse report *i*, an AE event rate over the report interval is *N/dt*. A rolling estimate of pulse rate, *R,* is maintained by applying a low pass filter with a configured cut-off frequency *f*, as follows: *R_i_ =* α *R_i-_*_1_ *+* (α – 1) *N/d* , where α = *e^-f dt^*.

The effect is an AE event rate signal which peaks when an event occurs and decays exponentially at a configurable rate, which is a key tuning parameter for AE rate control. The same form of low pass filter is also applied to actuator pressure and displacement signals. Our software implements conventional PID control and can be configured to control for hydraulic flow rate, actuator pressure, actuator displacement, cumulative AE count rate, estimated AE event rate or the axial strain of the sample. The controller can operate with multiple modes, each implementing a constant set-point or a linear ramp, and each mode has its own PID tuning parameters, established by empirical test. Each mode has a condition which permits the controller to advance to the next mode at a set threshold. A typical experiment uses several control modes to build up to a point where AE control can take over. The output of our controller drives the stepper motor of the syringe pump supplying hydraulic pressure to the actuator.

Two additional Cetoni Nemesys high-pressure syringe pumps were used to control the pore fluid pressure and confining fluid pressure of the sample. These were controlled separately using Cetoni’s QmixElements software to maintain constant fluid pressures during the experiment.


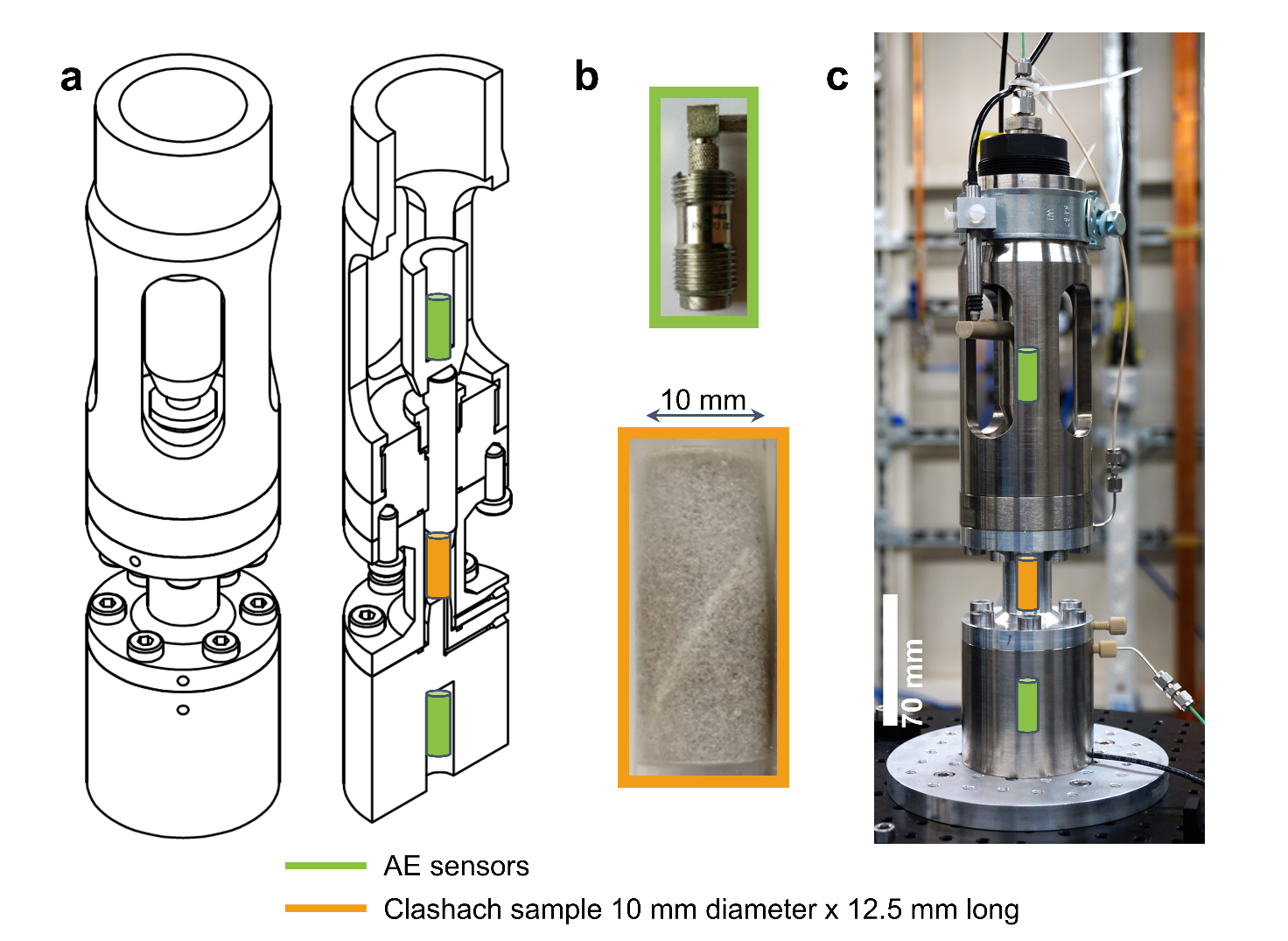


Supplementary Fig. 1. Our x-ray transparent rock deformation cell.

**a** Schematic and **c** photograph of our x-ray transparent rock deformation cell, Stór Mjölnir, with acoustic emissions (AE) monitoring. **b** Clashach sandstone sample (bottom; orange box) sits in the pressure vessel, and two piezoelectric transducers (top; green box) sit at either end of each piston, as shown in **a** and **c**. Axial displacement was measured outside the pressure vessel with a linear variable displacement transducer (LVDT).


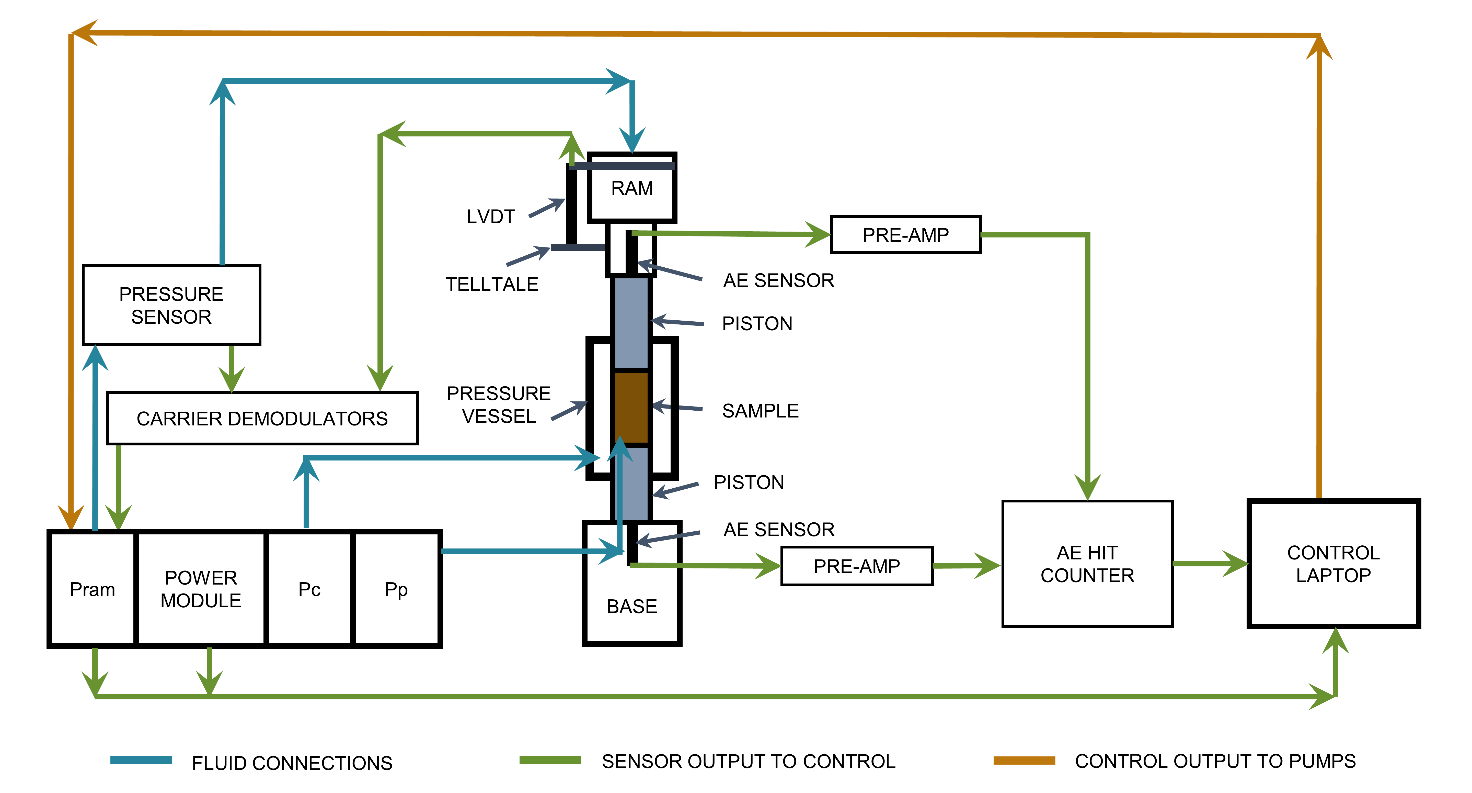


Supplementary Fig. 2. Experimental set-up.

Schematic of the experimental set-up, showing the pressurising fluid connections (blue), the Cetoni Base 600 power module and Nemesys syringe pumps for the ram, confining and pore fluids, sensor outputs (green) to the laptop operating the data logging and feedback control software, and the feedback control output (orange) to the ram pressure pump.


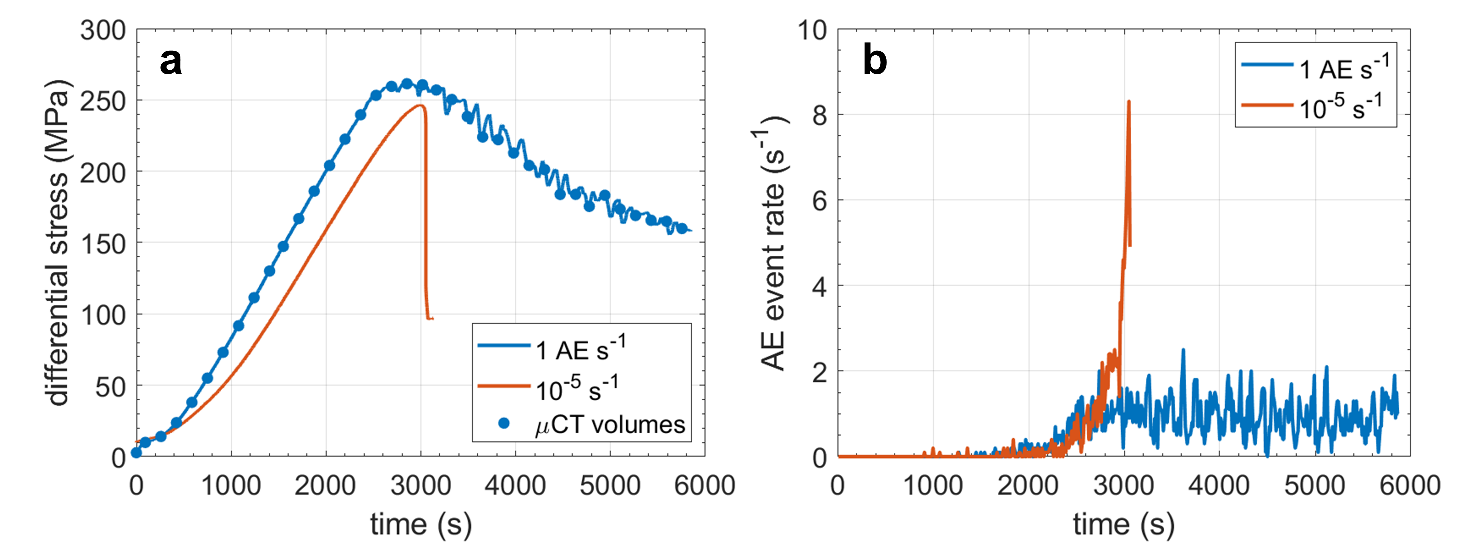


Supplementary Fig. 3. Comparison between experiments with and without acoustic emissions feedback control.

Evolution of **a** differential stress and **b** acoustic emissions (AE) event rate with time in Clashach sandstone both with AE feedback control (blue) and without (orange). In both cases the sample was loaded at a constant strain rate of 10^-5^ s^-1^ to start with. Without AE feedback (orange), the sample continued to be deformed at this constant strain rate until dynamic failure occurred following a rapid acceleration in AE events around peak stress. With AE feedback (blue), the constant AE event rate control took over at approximately 2400 s, once the desired AE event rate (1 AE s^-1^) had been reached; shortly before peak stress. The samples underwent triaxial deformation to brittle failure along a localised shear fault, evident from a rollover at peak stress in the axial pressure evolution followed by either a sudden drop in ram pressure (orange), representing dynamic macroscopic failure, or a gentle decrease in ram pressure (blue), representing stable fault propagation.


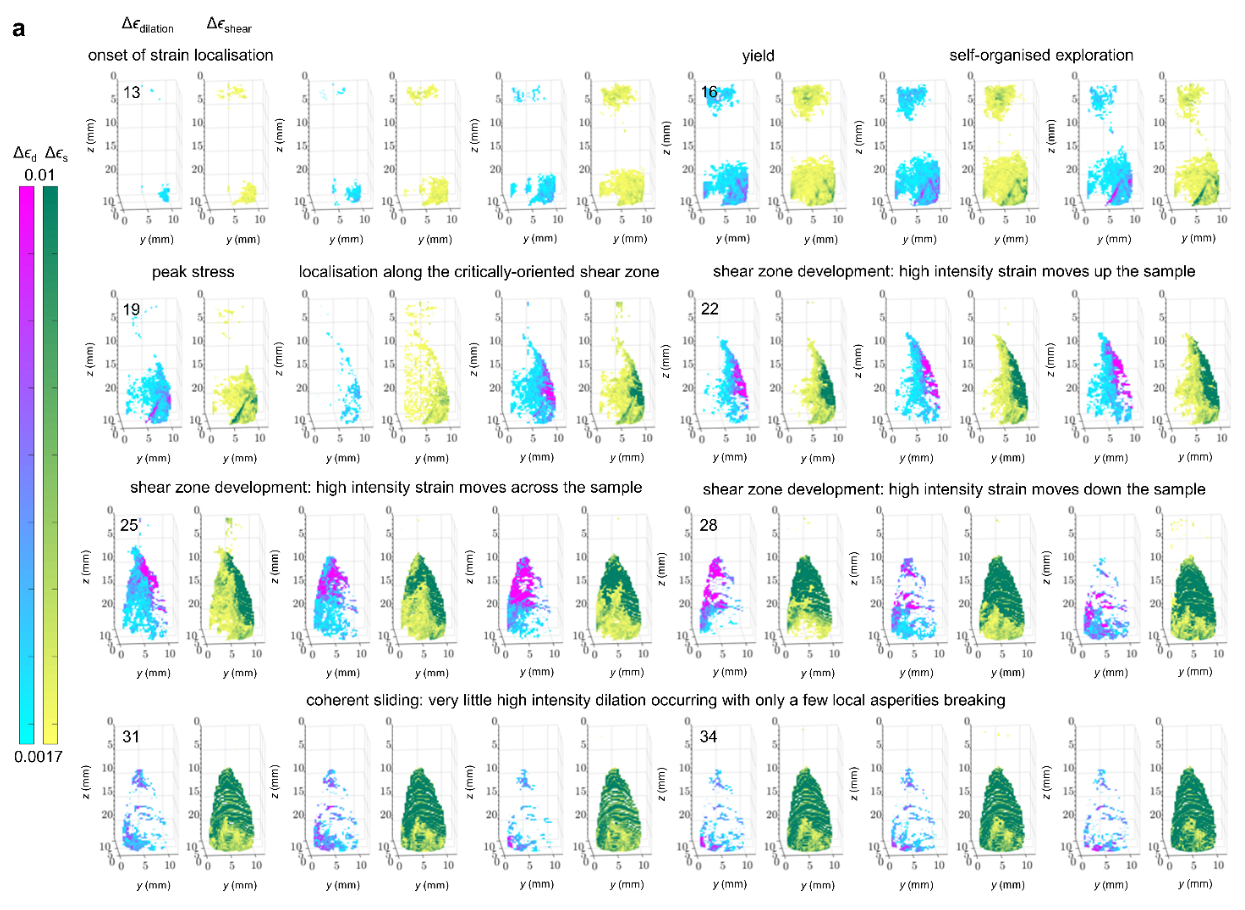

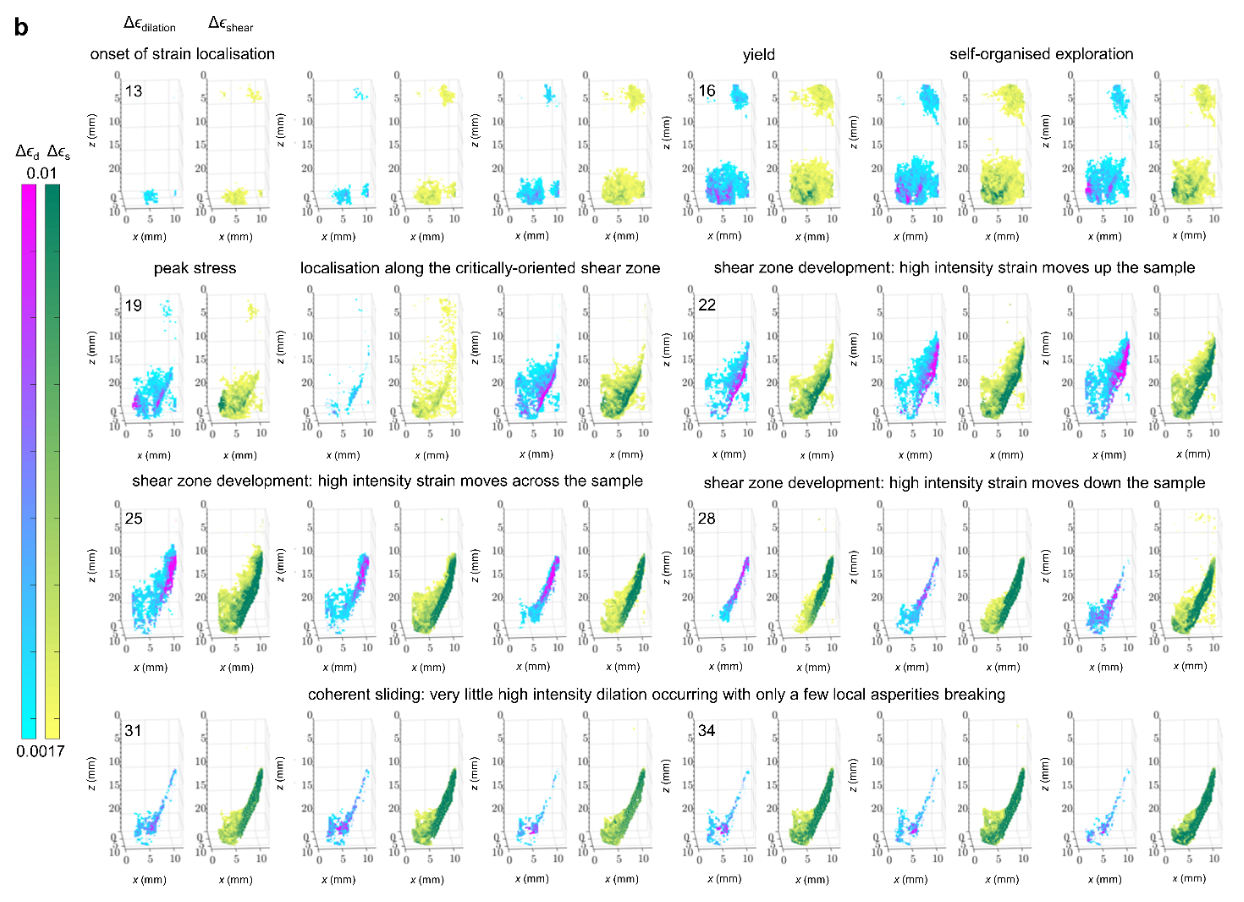


Supplementary Fig. 4. Local incremental dilation and shear strain fields.

Local incremental dilation, Δ*ϵ*_d_ (blue-pink), and shear, Δ*ϵ*_s_ (yellow-green), strain fields **a** parallel to strike and **b** perpendicular to strike for every strain increment from the onset of strain localisation to the end of loading. The lower threshold of 0.0017 was set at four standard deviations from the mean of the deviatoric (shear) strain error distribution (Supplementary Fig. 6) and the upper threshold shows regions with strain >0.01 (maximum Δ*ϵ*_s_ and Δ*ϵ*_d_ were ~0.04; Supplementary Figs. 4 and 5). The thresholds were chosen to visually highlight regions of localised strain. Number labels correspond to those in Figs. 1 and 2, with the strain increment between the numbered tomogram and its subsequent neighbouring tomogram.


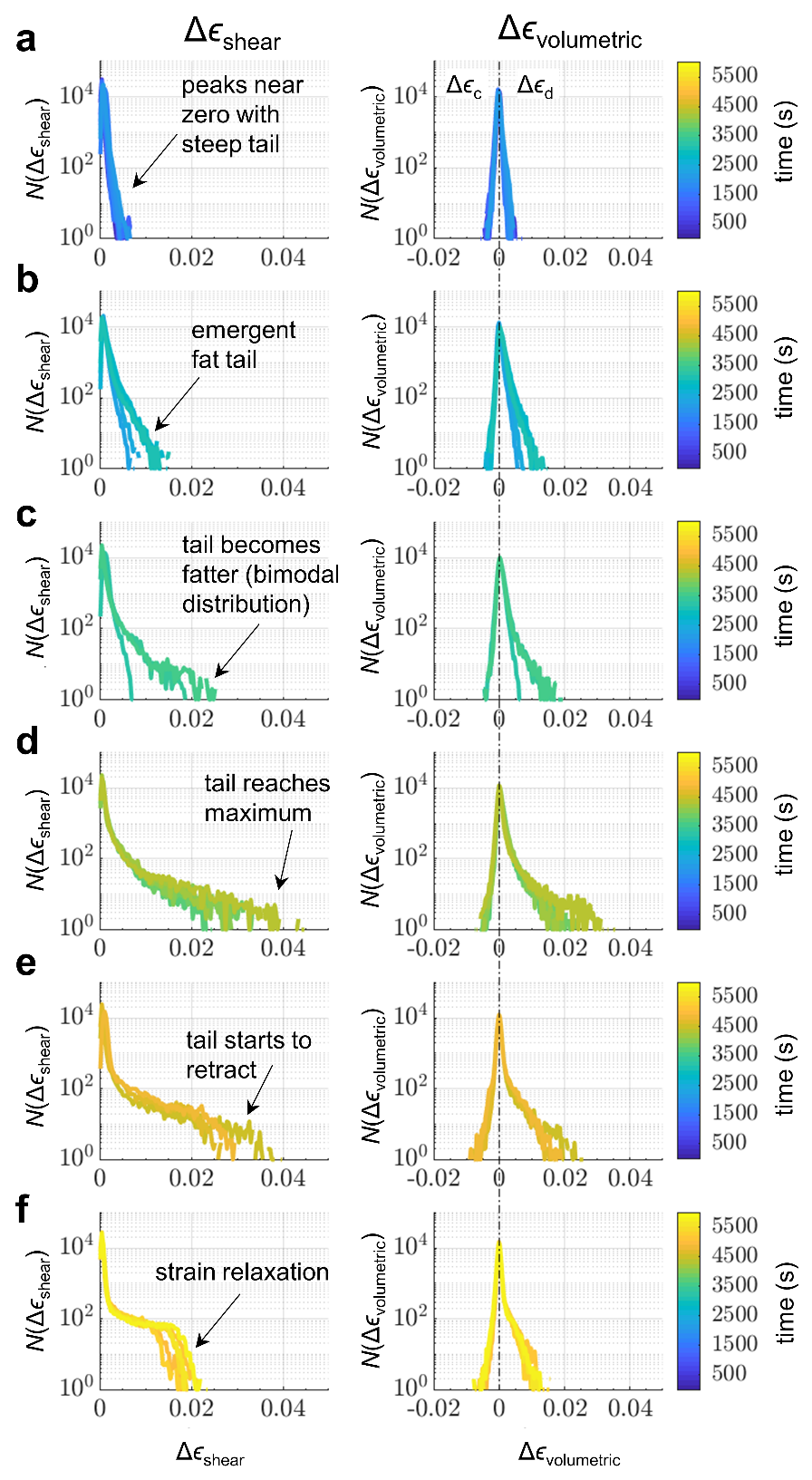


Supplementary Fig. 5. Local incremental strain field histograms.

Local incremental strain field histograms for **a** stage (i); up to scan a, exponential distribution peaked near zero with a steep tail showing equal partition between dilation (Δ*ϵ*_d_), compaction (Δ*ϵ*_c_) and shear (Δϵ_dev_) strain; **b** stage (ii); scans a-b, a fat tail emerges in both dilation and shear strain distributions (compaction remains the same) as strain localizes on the candidate strain clusters; **c** stage (iii), scans b-c, emergent tail becomes more pronounced as dilation and shear strain distributions become bimodal (break in slope apparent) once damage irreversibly localizes on the optimally-oriented cluster; **d** early stage (iv); scans c-e, tail reaches maximum extent as the shear zone propagates up and across the sample to become sample-width, and there is small amount of additional compaction; **e** late stage (iv); scans e-f, tail starts to retract (strain relaxation) as the sample-width shear zone propagates down the sample; **f** stage (v); scans f-end, the retraction is fully realized with a significant drop-off in the tail once the whole shear zone slides coherently.


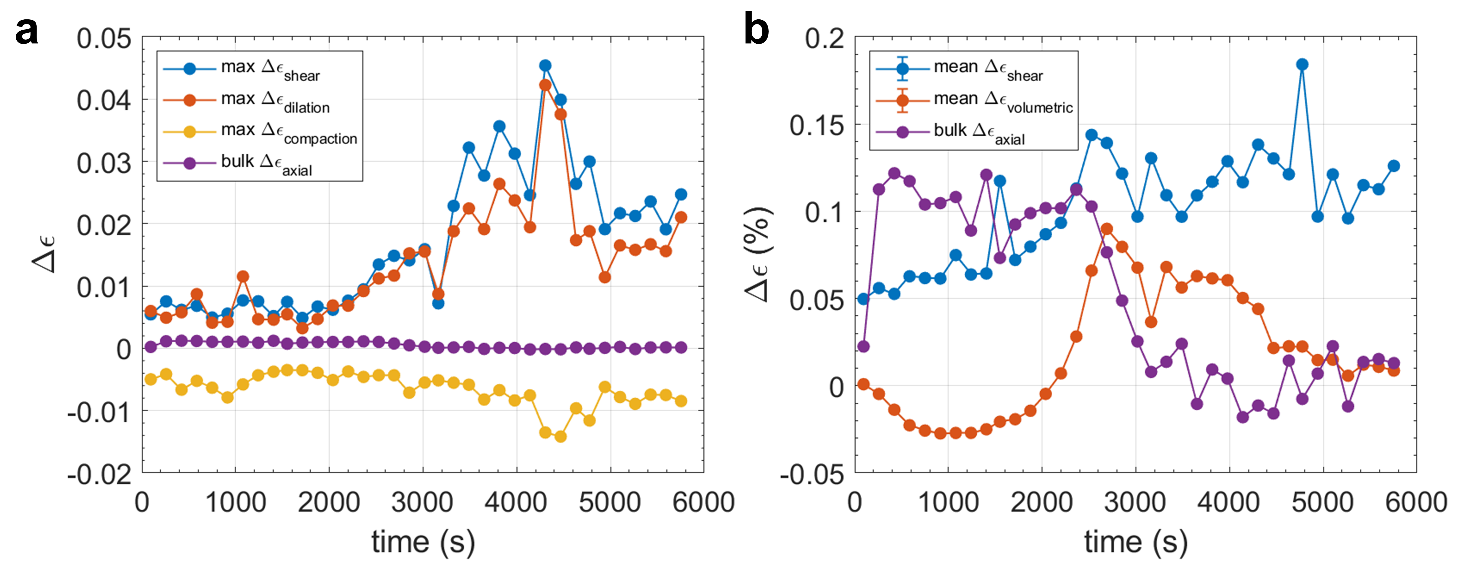


Supplementary Fig. 6. Evolution of local strain partition.

**a** Evolution of maximum local strain partition (shear strain in blue; dilation in orange; compaction in yellow) with reference to bulk axial strain (purple), showing how the maximum local strains, particularly shear strain and dilation, deviate significantly from the bulk axial strain after peak stress and during shear zone localisation and propagation. Refer to Fig. 1 in the main text for timings of the different stages. Strain on sample boundaries (bulk axial strain; purple) is much smaller than the maximum local strain on the shear zone. Although the acoustic emission (AE) rate feedback control reduced the strain rate by two orders of magnitude compared with the elastic loading phase, maximum local strains from peak stress onwards are larger than the bulk axial strain measurements by 2-2.5 orders of magnitude, indicating a large local amplification effect compared to the observed boundary strain (which mainly reflects the boundary loading conditions). Maximum compaction, dilation and shear strain are all similar at the start but then dilation and shear strains grow very large with slightly more shear strain than dilation, while compaction remains small even for this fairly porous sandstone. A small degree of anti-correlation between compaction and shear/dilation is apparent as the shear zone propagates down the sample and there is some degree of compaction localisation along the shear zone. **b** Evolution of mean local strain (shear strain in blue; volumetric strain in orange) with reference to bulk axial strain (purple), showing how, on average, initial, diffuse compaction is eventually swamped by dilation coming from increasingly localized mechanisms. Mean shear strain, on the other hand, increases steadily throughout the experiment.


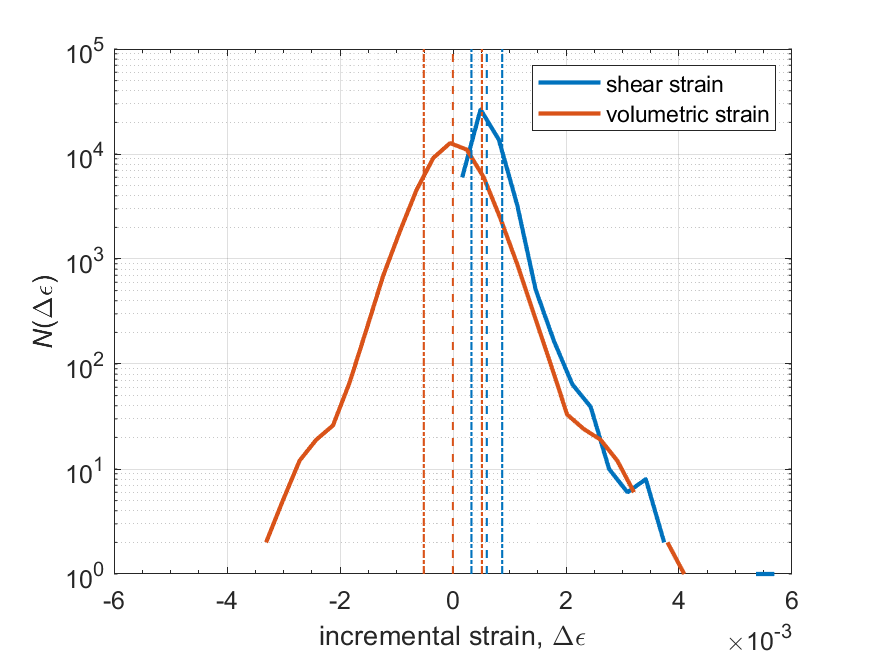


Supplementary Fig. 7. Frequency distribution of digital volume correlation error.

Frequency distribution of digital volume correlation (DVC) error, calculated from DVC between two μCT volumes scanned at identical pressure conditions prior to loading. The mean error in the volumetric strain (dotted blue line) is -2.04 x10^-6^, with one standard deviation of 5.14 x10^-4^ (dash-dot blue line). The mean error in the deviatoric (shear) strain (dotted orange line) is 5.98 x10^-4^, with one standard deviation of 2.72 x10^-4^ (dash-dot orange line). Dilation is defined as positive volumetric strain and compaction is defined as negative volumetric strain. For a given noise in the displacement field, the contribution of numerical noise in the calculation of the first and second strain invariants is higher for volumetric strains (first invariant) than deviatoric strains (second invariant). However, our estimate of DVC noise here shows that the error distributions are comparable between the two. In fact the strain value at one standard deviation from the mean is slightly smaller for volumetric strains (±0.0005) than for deviatoric strains (0.0009), although the volumetric error standard deviation is twice that of the deviatoric error. This indicates that the numerical noise is smaller than other sources of noise in the strain measurement, although the sources of such noise are not immediately obvious. It is also evidence that our observed large strains are meaningful and discernible above the noise (the largest observed strain values being an order of magnitude larger than the largest observed error values).


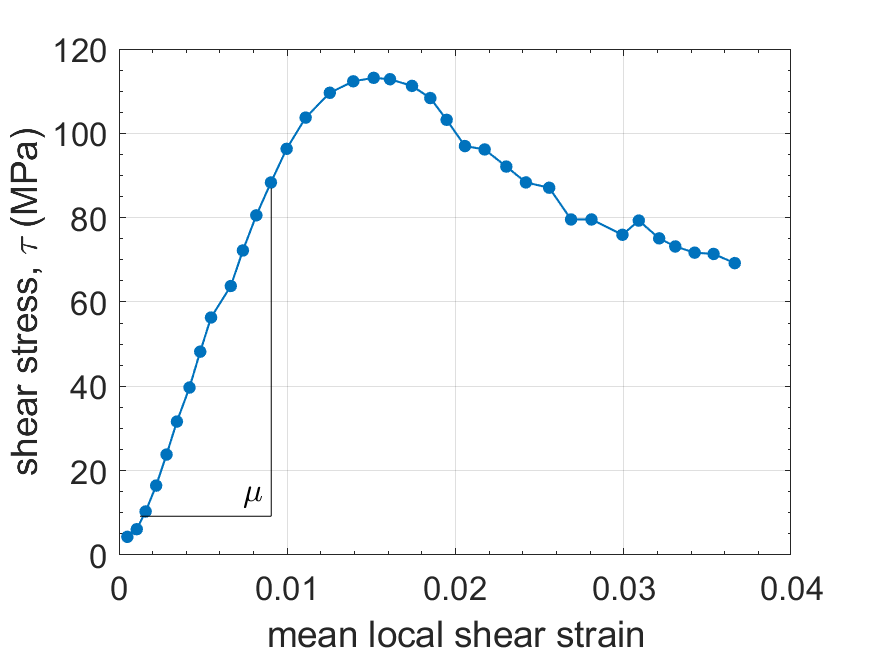


Supplementary Fig. 8. Shear stress evolution with local shear strain.

Shear stress vs mean local shear strain at each x-ray microtomographic (μCT) volume (blue dots) for estimation of intact shear modulus of the sample, *μ*, over the data range shown. *μ* = 10.45 ± 0.6 GPa.


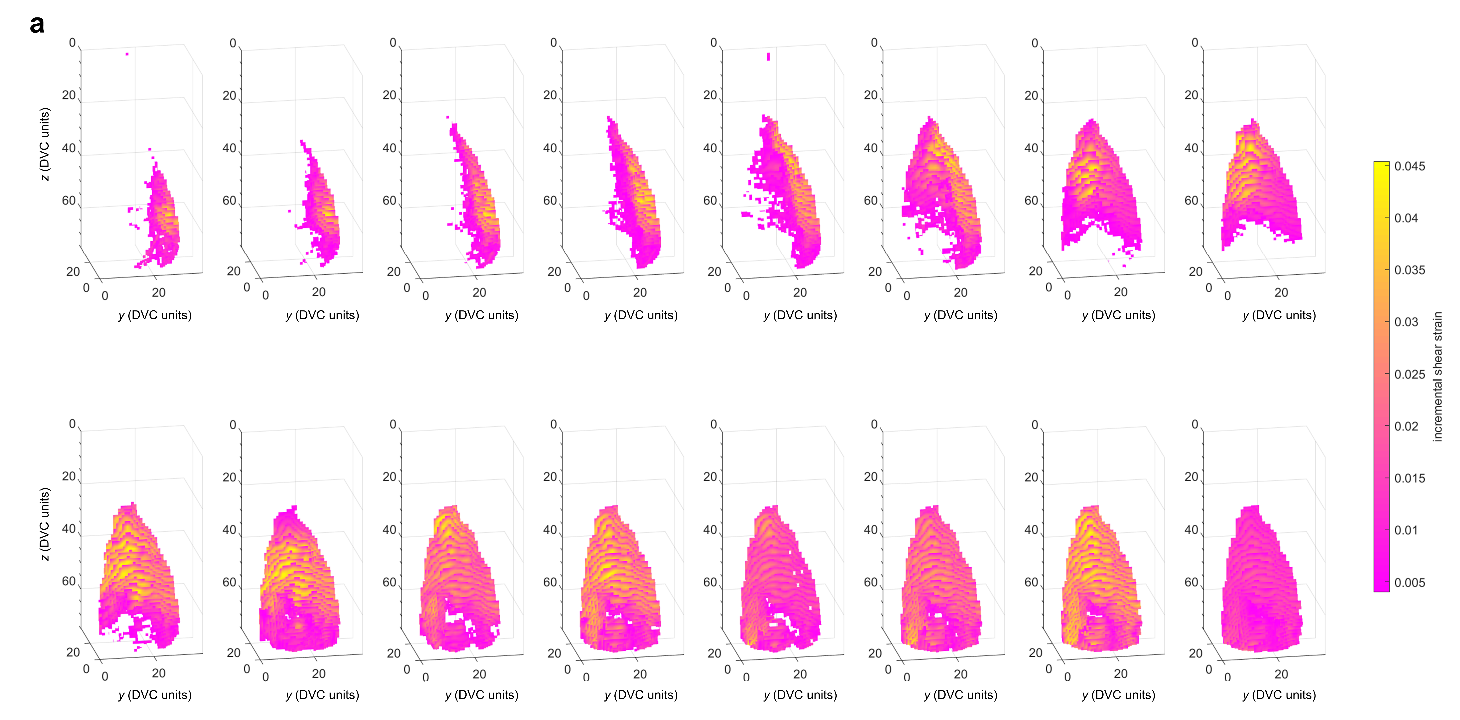


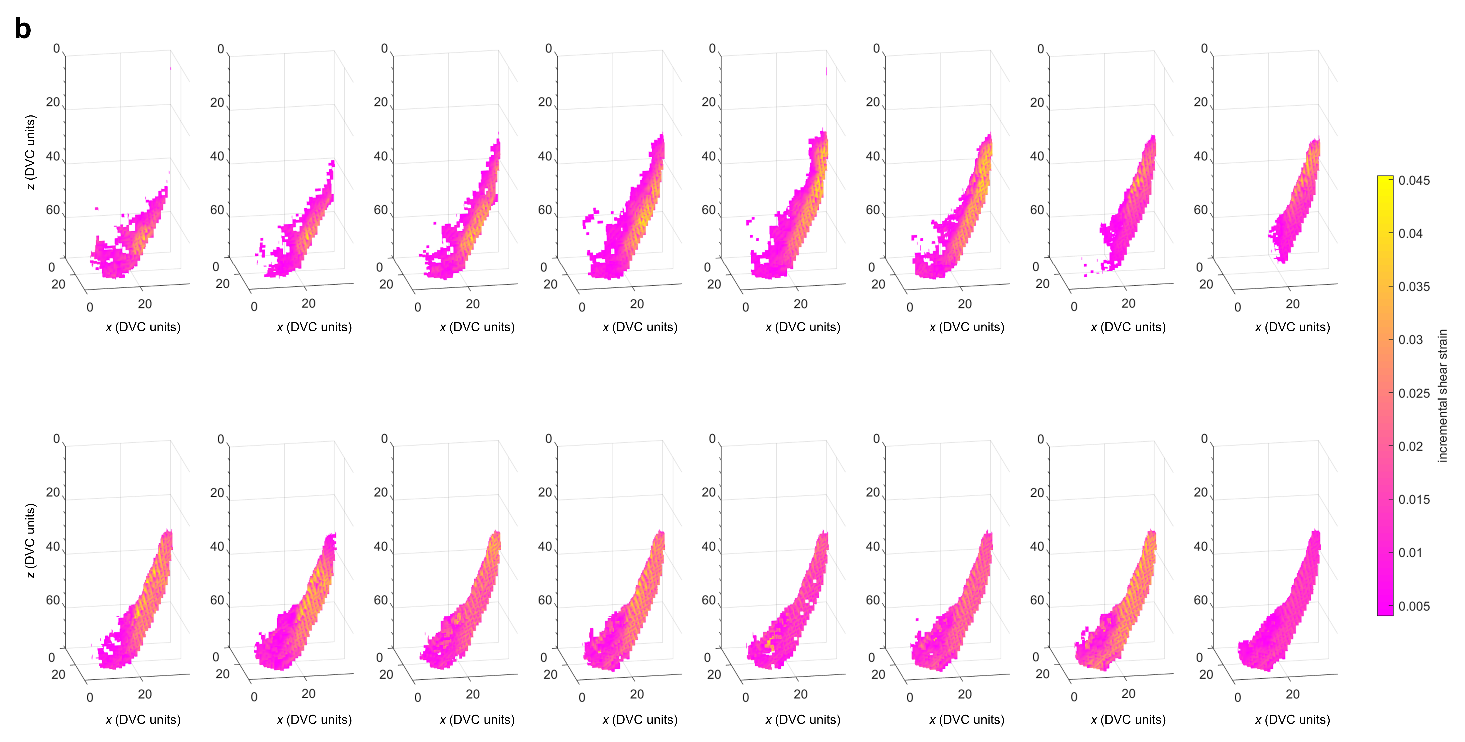


Supplementary Fig. 9. Connected shear zone object.

Connected shear zone object, as defined from the shear strain fields, shown from irreversible localisation (strain increment 21) to end of loading **a** parallel to strike and **b** perpendicular to strike for estimation of *θ* and $\bar{{\Delta\epsilon}_{shear}}$ by approximating a best-fitting ellipse. The axes scales are in digital volume correlation (DVC) window lengths, with 1 window length = 316.4 μm (~1 grain size).


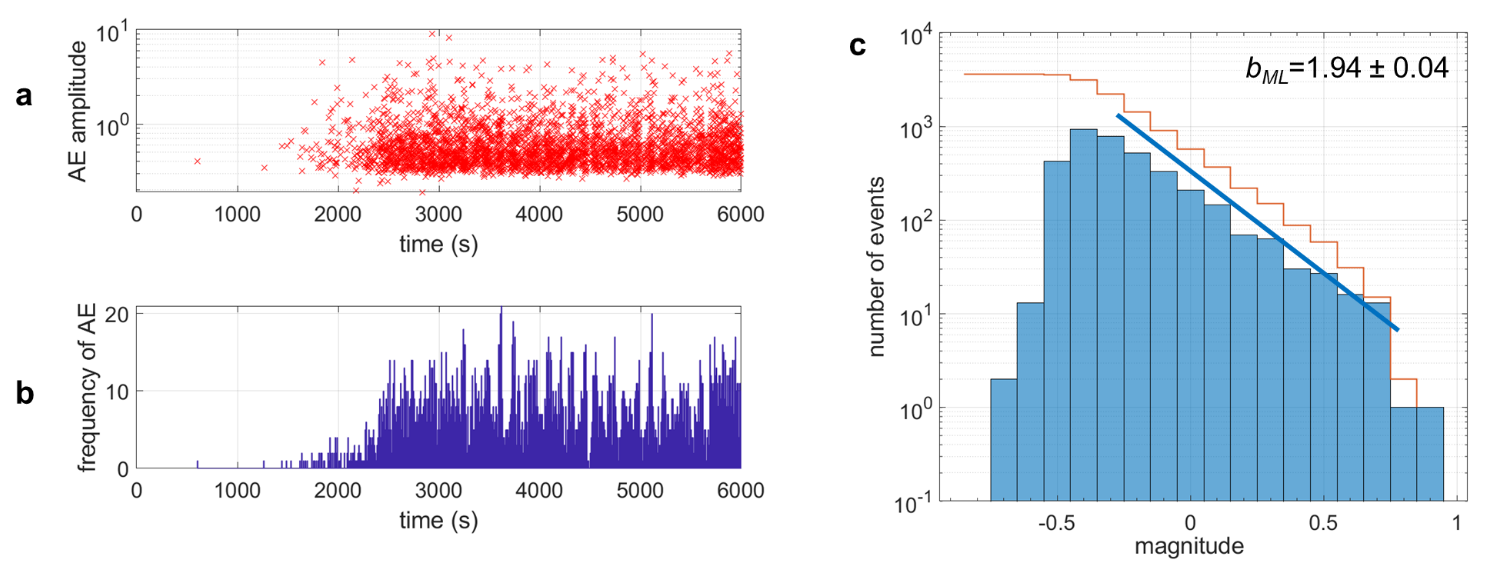


Supplementary Fig. 10. Evolution of acoustic response.

**a** Acoustic emission (AE) amplitude as a function of time, **b** AE frequency as a function of time defined in 10 s windows, and **c** frequency-magnitude plot showing incremental (blue bars) and cumulative (orange line) distributions for all detected events throughout the experiment (i.e., all those detected by 6000 s at the point of bulk sample failure). Magnitude is the log of the amplitude, which was estimated as the maximum of the Hilbert envelope of the AE waveform. The Gutenberg-Richter *b*-value was estimated to be 1.94 with 95% confidence.


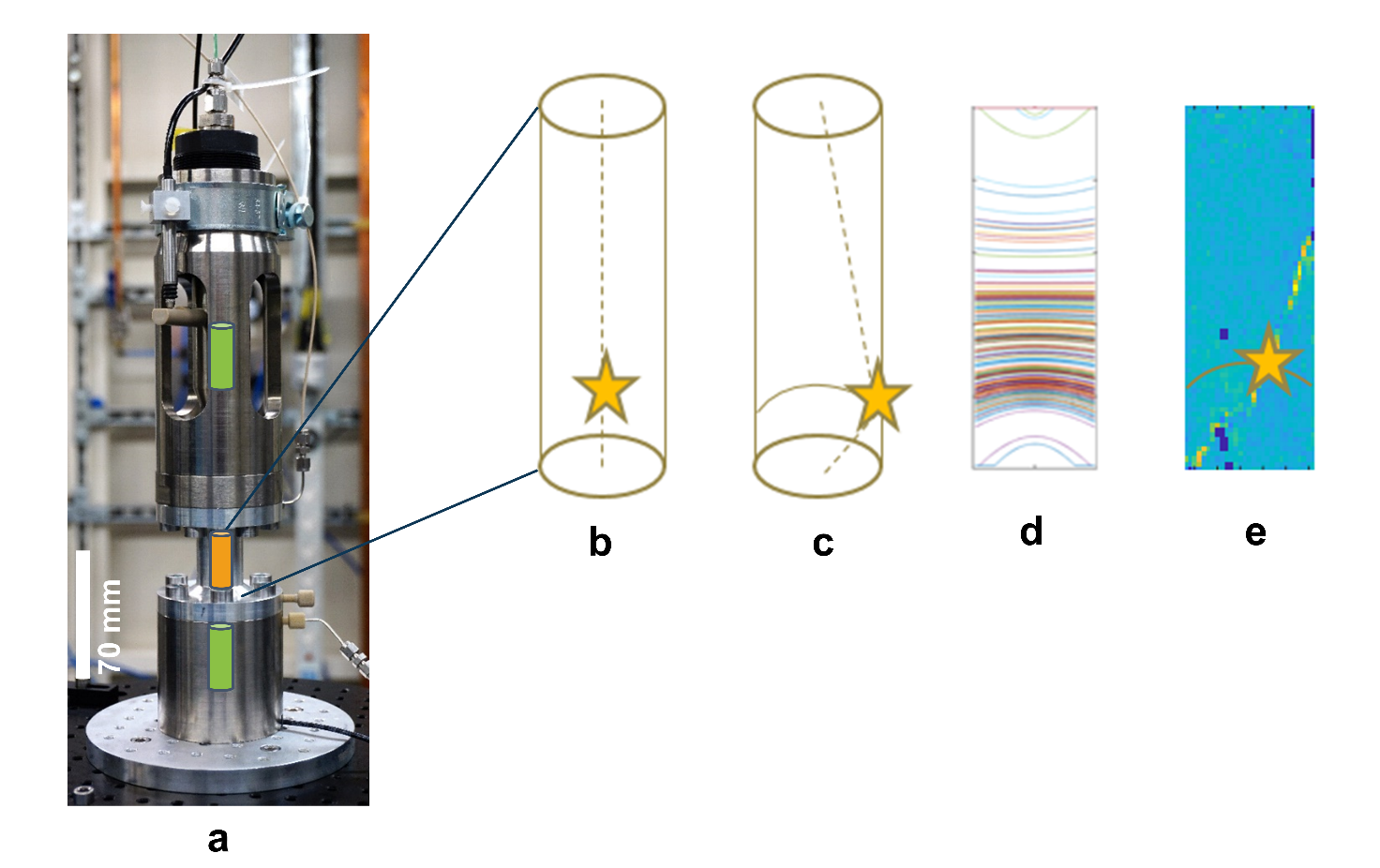


Supplementary Fig. 11. Acoustic emission location constrained by kinematics and local strain.

**a** Experimental setup showing location of seismic sensors (green) and sample (orange). **b**-**c** Acoustic emission (AE) location constraint by kinematics: the relative time-delay of each AE arrival recorded at the top and bottom sensor can be used to trace a circular hyperboloid (kinematic constraint) with two potential position radii shown at **b** zero and **c** maximum radius. **d** Circular hyperboloids for all AEs: flatter/more curved when the relative time-delay is close to zero/large implying AE location near the middle/end of the sample, respectively. **e** AE location constraint by local strain: Assuming that each AE occurred at the largest local strain within its circular hyperboloid, a unique position can be defined.


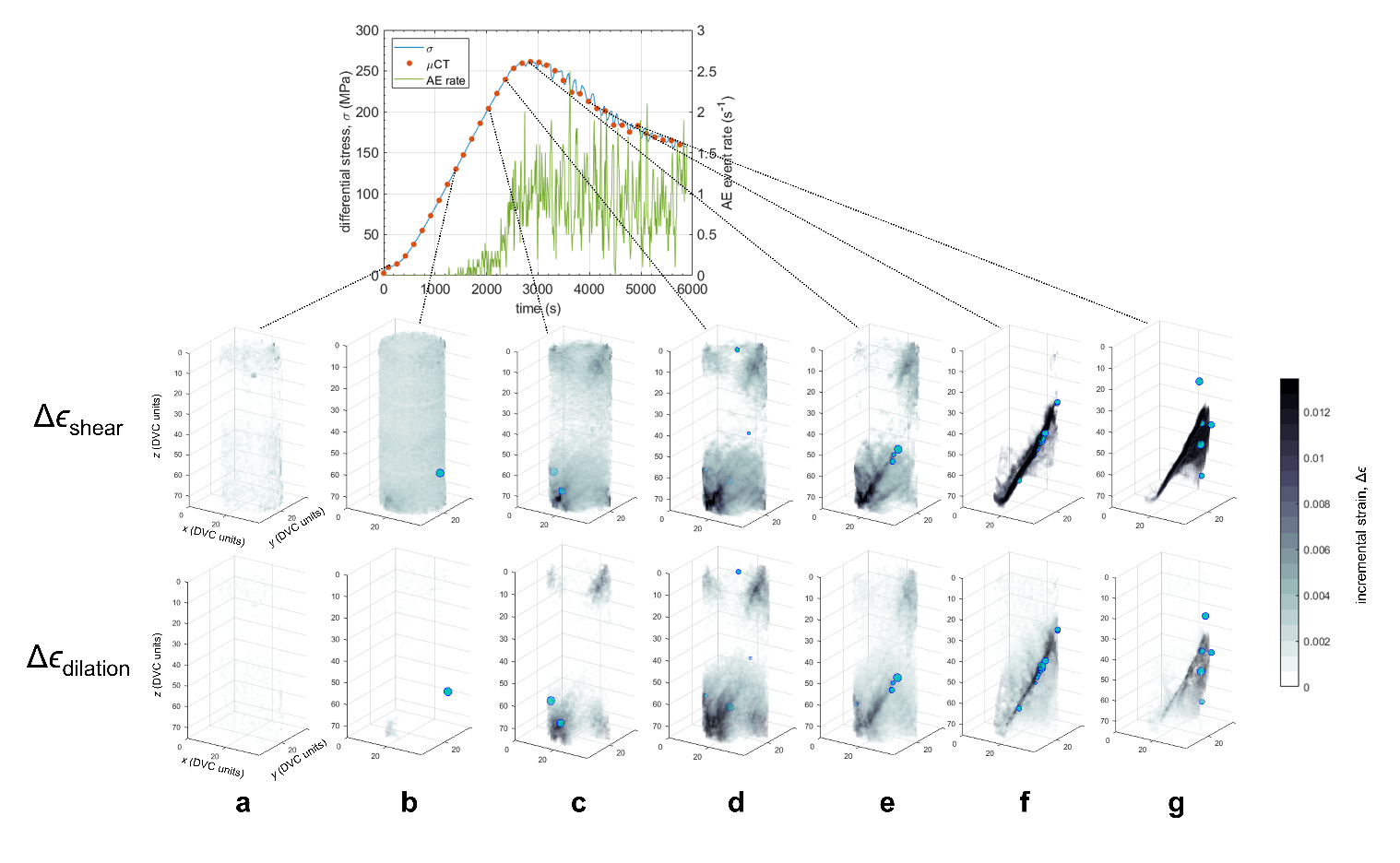


Supplementary Fig. 12. Evolution of acoustic emissions locations with local incremental strain and differential stress.

Evolution of differential stress and acoustic emission (AE) event rate (top), incremental shear strain (middle) and incremental dilation (bottom), with AE locations shown as bright blue circles (sizes show relative amplitudes). **a**-**g** Local incremental 3D strain between the x-ray microtomographic (μCT) volume from the time shown (orange dots) and the previous μCT volume: **a** at the onset of deformation, where strains are low and there are no AE, **b** with further deformation AE start to occur, **c** at the yield point, **d** during the strain hardening stage; cracking picks up and larger AE occur, **e** at peak differential stress deformation starts to localize, **f** during the strain softening phase, the shear zone forms, **g** the shear zone becomes sample-sized. The axes scales are in digital volume correlation (DVC) window lengths, with 1 window length = 316.4 μm (~1 grain size). Overall, the relative amplitude of AE is not correlated with local strain intensity. A movie of AE location evolution with volumetric strain is given in Supplementary Movie 9.


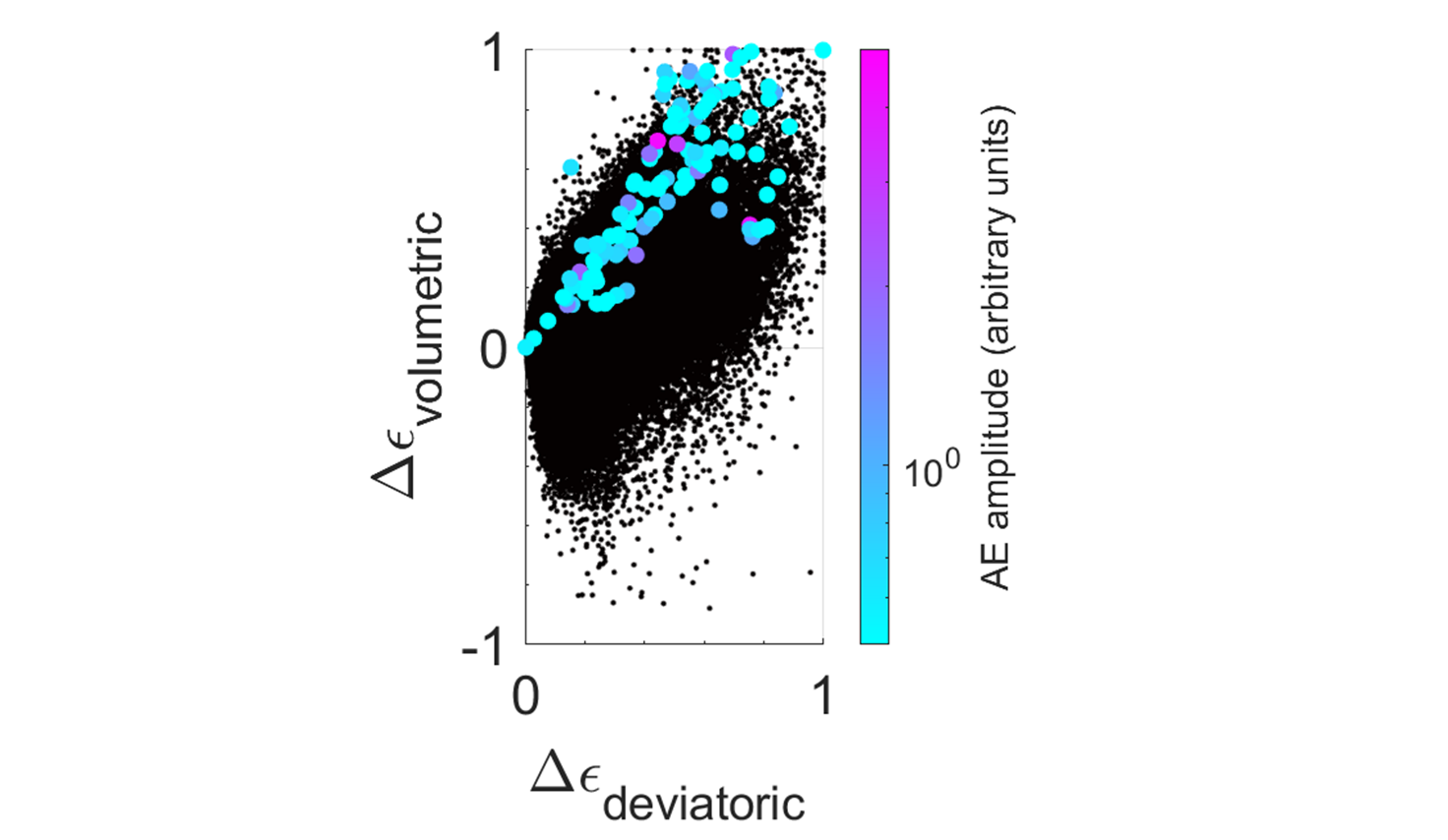


Supplementary Fig. 13. Correlation between local incremental volumetric and shear strain.

Incremental deviatoric (shear) strain vs. incremental volumetric (with dilation defined as positive) strain, each normalized to their respective maximum, for the whole experiment. Locations of acoustic emission (AE) events are shown as coloured dots showing a strong correlation of deviatoric vs. volumetric strain. The colour scale at the AE locations shows AE amplitude in log scale, estimated from the maxima of the Hilbert envelopes of the AE waveforms.


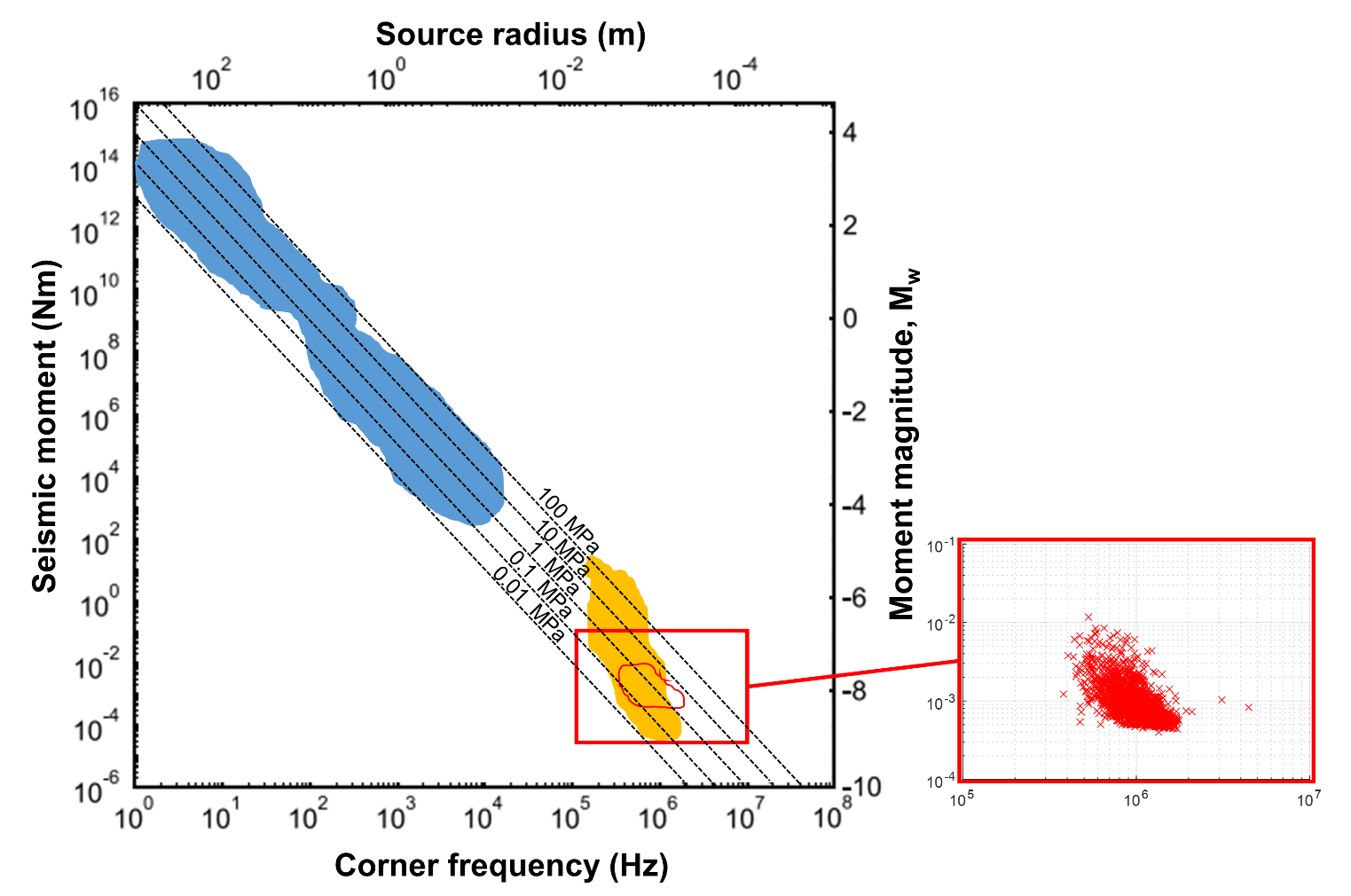


Supplementary Fig. 14. Relationship between source parameter relations and stress drop for our data, compared with previous studies.

Stress drop and source parameter relations using the Madariaga^1^ source model for our data shown by the red crosses magnified in the red inset, compared with 23 previous field (natural-, mining induced- and fracking induced seismicity) and laboratory (acoustic emission events) studies shown as blue and orange shaded areas respectively. The data from these studies are presented in Blanke^2^ et al., Fig. 2a and Kwiatek^3^ et al., Fig. 8. Dashed lines indicate constant stress drops of 0.01–100 MPa.


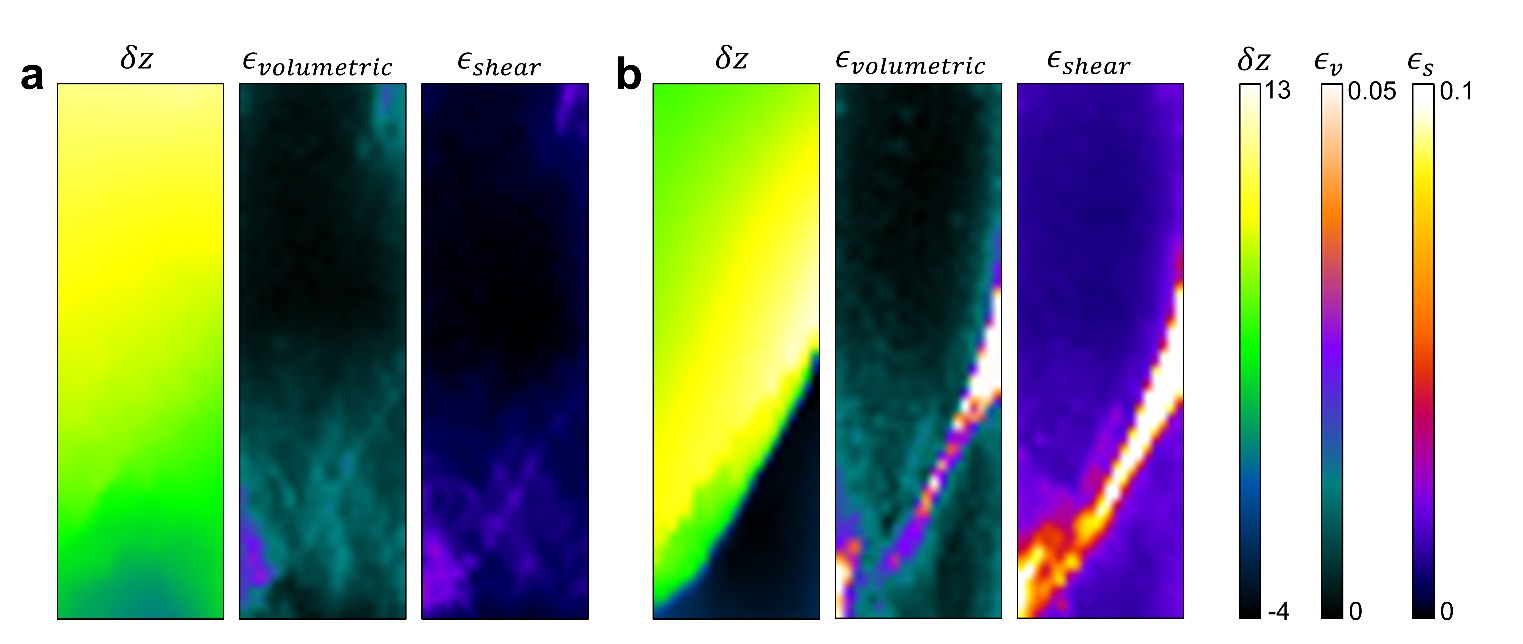


Supplementary Fig. 15. Median projections of vertical displacement, volumetric and shear strains before and after peak stress.

Median projections perpendicular to strike of vertical displacement ($\delta z$), volumetric strain ($\epsilon_{volumetric}$) and shear strain ($\epsilon_{shear}$), summed over strain increments **a** 14-19 approaching peak stress, and **b** 20-36 after peak stress. This figure highlights the influence of the region of enhanced vertical compaction in the bottom left part of the sample in **a** leading to localised dilation (tensile micro-cracking) and shear strain just below that region. This weakening in the microstructure facilitated bulk left-lateral motion, leading to strain localisation along and subsequent development of the critically-oriented shear band, as seen in **b**.


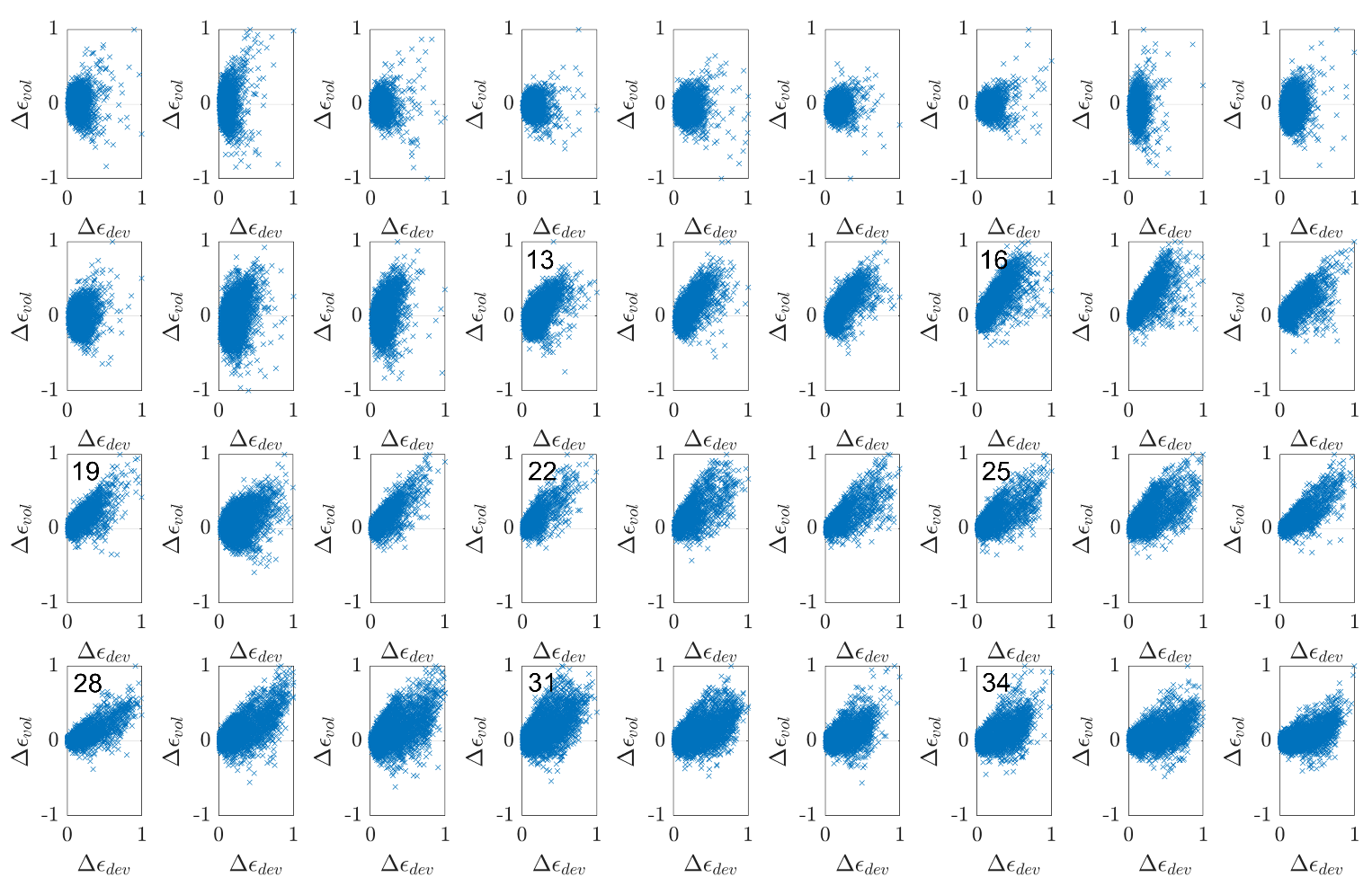


Supplementary Fig. 16. Local incremental volumetric and shear strain cross-plots for the full time-series.

Incremental strain cross-plots for the full time-series, showing incremental deviatoric (shear) strain, $\epsilon_{dev}$, at each location (digital volume correlation window) within the sample, plotted against the incremental volumetric strain, $\epsilon_{vol}$ at the same location. Dilation is defined as positive volumetric strain. Numbers relate to the tomogram numbers and strain field increments shown in Figs. 1 and 3. Shear strain values were normalised to the maximum shear strain in each increment. Volumetric strain values were normalised to the absolute maximum volumetric strain, which was compaction only in four strain increments (3, 5, 6 and 11), most prior to the onset of acoustic emissions (increment 9), and all prior to the onset of strain localisation (increment 13).


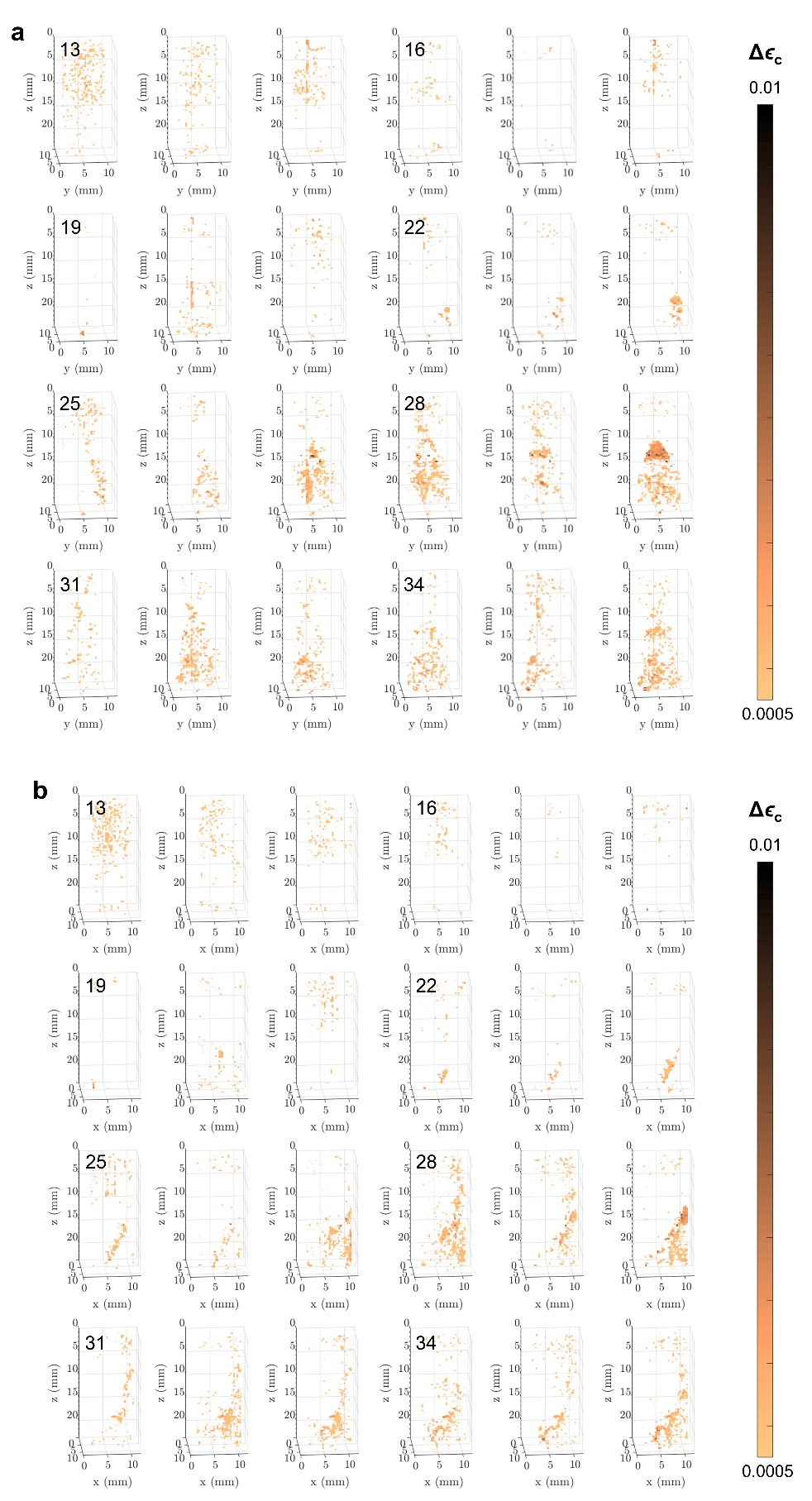


Supplementary Fig. 17. Local incremental compaction fields.

Full time-series of 3D incremental absolute compaction fields from the onset of strain clustering (marked in Fig. 1c). Incremental compaction, Δ*ϵ*_c_ (yellow-red) was calculated from digital volume correlation (DVC) between successive pairs of x-ray microtomographic (μCT) volumes and are shown **a** parallel to strike (*y,z* orientation) and **b** perpendicular to strike (*x,z* orientation). The lower threshold of 0.0005 was set at one standard deviation from the mean of the volumetric strain error distribution in the negative direction (Supplementary Fig. 7) and the upper threshold shows regions with strain >0.01 (maximum Δ*ϵ*_c_ was ~0.015; Supplementary Figs. 5 and 6). The thresholds were chosen to visually highlight regions of localised strain. Number labels correspond to those in Figs. 1, 2 and 3, with the strain increment between the numbered tomogram and its subsequent neighbouring tomogram.

Supplementary Table 1. Shear zone orientation and off-fault : on-fault dilation and shear strain.

Angle, *θ*, between the loading axis and the shear zone orientation estimated from the best-fit ellipse of the shear zone object identified in each 3D incremental shear strain field. The scan increment numbers are relative to the start of the experiment and start from the increment following the increment after peak stress. Mean observed *θ* = 30.3° ± 8.4°. Also given is the proportion of off-fault dilation and shear strain relative to on-fault shear strain, Δ*ϵ*_off_/Δ*ϵ*_on_. This shows that during strain softening, just before final localisation, almost one third of the shear strain occurs off-fault. This decreases to 13% during final localisation (scan increment 21-22) and then to 6% by the end of the test. In contrast, less than 10% of dilation occurs off-fault. This decreases from 9% during final localisation to 1% by the end of the test.

| scan increment | *θ* (°) | shear strain | dilation |
| --- | --- | --- | --- |
|  |  | Δ*ϵ*_off_/Δ*ϵ*_on_ | Δ*ϵ*_off_/Δ*ϵ*_on_ |
| 20-21 | 34.356 | 0.27 | 0.07 |
| 21-22 | 38.344 | 0.13 | 0.09 |
| 22-23 | 34.136 | 0.10 | 0.07 |
| 23-24 | 30.733 | 0.10 | 0.07 |
| 24-25 | 29.849 | 0.08 | 0.06 |
| 25-26 | 30.274 | 0.10 | 0.06 |
| 26-27 | 31.562 | 0.09 | 0.05 |
| 27-28 | 26.614 | 0.07 | 0.02 |
| 28-29 | 21.565 | 0.06 | 0.01 |
| 29-30 | 28.588 | 0.06 | 0.02 |
| 30-31 | 30.494 | 0.10 | 0.01 |
| 31-32 | 28.973 | 0.07 | 0.02 |
| 32-33 | 29.797 | 0.07 | 0.01 |
| 33-34 | 29.595 | 0.07 | 0.01 |
| 34-35 | 29.933 | 0.07 | 0.01 |
| 35-36 | 30.088 | 0.06 | 0.01 |
| 36-37 | 30.184 | 0.06 | 0.01 |

Supplementary Table 2. Estimate of rotational slip from crack rotation markers.

Crack rotation markers to estimate feasible proportion of rotational slip with respect to the total observed local slip, Δ*u* (Fig. 5b). Rotational slip was calculated using the cosine rule, and was, on average, 77 ± 29% of local Δ*u*.

| crack # | rotation angle (°) | initial crack length (mm) | final crack length (mm) | rotational slip (mm) | local slip, Δ*u* (mm) | rotational slip / relative slip |
| --- | --- | --- | --- | --- | --- | --- |
| 1 | 25.6 | 0.173 | 0.153 | 0.075 | 0.104 | 0.72 |
| 2 | 54.2 | 0.187 | 0.128 | 0.153 | 0.121 | 1.26 |
| 3 | 27.4 | 0.121 | 0.101 | 0.056 | 0.104 | 0.54 |
| 4 | 36.6 | 0.128 | 0.138 | 0.084 | 0.146 | 0.58 |
| average | 35.9 | 0.152 | 0.130 | 0.090 | 0.119 | 0.77 |

Supplementary References

1. Madariaga, R. Dynamics of an expanding circular fault, *Bull. Seismol. Soc. Am.* **66**(3), 639–666 (1976).
2. Blanke, A. B., Kwiatek, G., Goebel, T. H. W., Bohnhoff, M. & Dresen, G. Stress drop-magnitude dependence of acoustic emissions during laboratory stick-slip. *Geophys. J. Int.* **224**, 1371-1380 (2021).
3. Kwiatek, G., Plenkers, K., Dresen, G. & JAGUARS Research Group. Source parameters of pico-seismicity recorded at Mponeng Deep Gold Mine, South Africa: implications for scaling relations, *Bull. Seismol. Soc. Am.* **101**(6), 2592–2608 (2011).
