# Supplementary material for: Seismic events miss important kinematically governed grain scale mechanisms during shear failure of porous rock
Source: Nat Commun. 2022 Oct 18;13:6169. doi: 10.1038/s41467-022-33855-z (PMC9579157; doi:10.1038/s41467-022-33855-z)
Supplement: Supplementary file 3 — Description of Additional Supplementary Information [file 41467_2022_33855_MOESM3_ESM.pdf]

Description of Additional Supplementary Information

Title: Supplementary Movie 1

Description: Time-series of 2D x-ray microtomographic (μCT) slices showing the bottom two thirds of the sample and the full shear zone. For scale, grains are 0.25–0.4 mm in diameter.

Title: Supplementary Movie 2

Description: Time-series of 2D x-ray microtomographic (μCT) slices zoomed-in to the shear zone. For scale, grains are 0.25–0.4 mm in diameter.

Title: Supplementary Movie 3

Description: Evolution of shear strain across-strike (x,z-orientation) with lower threshold of 0.0005 (one standard deviation from the mean of the error in the volumetric strain – see Supplementary Fig. 7 – to allow comparison with diffuse, low amplitude compaction).

Title: Supplementary Movie 4

Description: Evolution of dilation across-strike (x,z-orientation) with lower threshold of 0.0005.

Title: Supplementary Movie 5

Description: Evolution of compaction across-strike (x,z-orientation) with lower threshold of 0.0005.

Title: Supplementary Movie 6

Description: Evolution of shear strain along-strike (y,z-orientation) with lower threshold of 0.0005.

Title: Supplementary Movie 7

Description: Evolution of dilation along-strike (y,z-orientation) with lower threshold of 0.0005.

Title: Supplementary Movie 8

Description: Evolution of compaction along-strike (y,z-orientation) with lower threshold of 0.0005.

Title: Supplementary Movie 9

Description: Evolution of acoustic emission (AE) locations (blue circles) and volumetric strain (positive strain is dilation). The axes scales are in digital volume correlation (DVC) window lengths, with 1 window length = 316.4 μm (~1 grain size).
